# Patent Mining on the Use of Antioxidant Phytochemicals in the Technological Development for the Prevention and Treatment of Periodontitis

**DOI:** 10.3390/antiox13050566

**Published:** 2024-05-03

**Authors:** Paulo José Lima Juiz, Luiza Teles Barbalho Ferreira, Edilson Araújo Pires, Cristiane Flora Villarreal

**Affiliations:** 1 Center for Science and Technology in Energy and Sustainability, Federal University of Recôncavo da Bahia, Feira de Santana 44042-280, BA, Brazil; limajuiz@ufrb.edu.br (P.J.L.J.); luiza@ufrb.edu.br (L.T.B.F.); 2 Faculty of Education and Integrated Sciences of Sertão de Canindé, State University of Ceará, Canindé 62700-000, CE, Brazil; edilson.pires@uece.br; 3 School of Pharmacy, Federal University of Bahia, Salvador 40170-290, BA, Brazil

**Keywords:** antioxidant, periodontal disease, phytochemical

## Abstract

Periodontal disease is an inflammatory condition characterized by an aberrant immune response against a dysbiotic dental biofilm, with oxidative stress performing an essential role in its pathogenesis. This paper presents a patent mining, performed in the Orbit Intelligence patent database, related to antioxidant phytochemicals in the technological developments that are working to prevent and treat periodontal disease. To access the documents, the descriptors “PERIODONTAL” and “ANTIOXIDANT” were typed in the title, abstract, and claim search fields. A total of 322 patents demonstrate the growing interest in researching natural antioxidants for scientific and technological purposes. The top ten countries regarding the number of family patents produced were the United States, the European Office, Japan, South Korea, China, India, Mexico, Denmark, Canada, and Great Britain. The most cited compounds were vitamin C, green tea, quercetin, melatonin, lycopene, resveratrol, and curcumin. These compounds have been used for the technological development of gels, membranes, dentifrices, chewing gum, orally disintegrating film, mouthwash, mouth spray, and mouth massage cream and exhibit the ability to neutralize free radicals and reduce oxidative stress, a critical factor in the development and progression of periodontal diseases. The patent documents have shown that using antioxidant compounds in conjunction with traditional periodontal treatments is a promising area of interest in periodontal therapy.

## 1. Introduction

Periodontal disease is a term used to describe a range of inflammatory conditions, including gingivitis and periodontitis, characterized by the progressive destruction of periodontal supportive tissues and mediated by an aberrant immune response against dysbiotic microbial communities [1,2]. Reactive oxygen species at inflammation sites have also been implicated in periodontal disease pathogenesis [3]. Therefore, controlling pathogenic bacterial colonization, local immune response, and oxidative stress might be fundamental to prevent tissue destruction.

**Figure 1:**
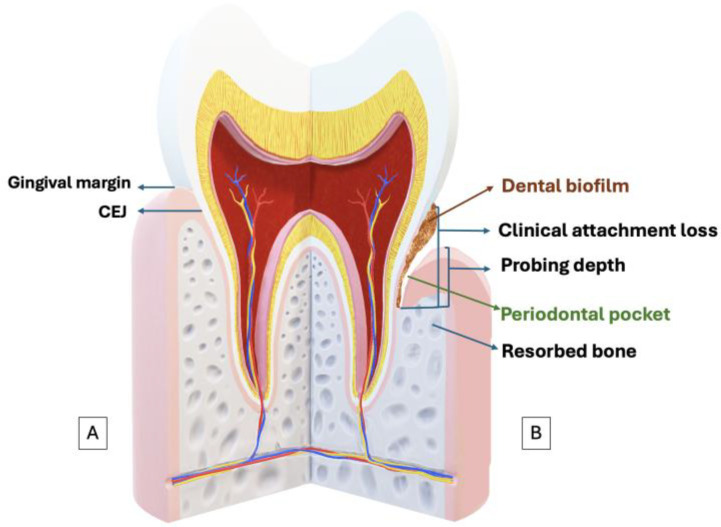
(**A**) Schematic representation of periodontal health. (**B**) Schematic representation of periodontal disease. Probing depth, the distance from the gingival margin to the apical portion of the gingival sulcus; the clinical attachment loss or clinical attachment level refers to the distance from the cemento-enamel junction (CEJ) to the apical extent (depth) of the periodontal sulcus, indicating how much tissue support has been lost.

Clinically, periodontal disease may be recorded as the loss of clinical attachment concerning the cemento-enamel junction (CEJ). Other symptoms include inflammation of the gums, characterized by erythema, swelling, and bleeding on probing. Additional signs may include the formation of periodontal pockets, recession of the gum margins, and involvement of furcation areas. Patients may also experience increased tooth mobility [4]. (Figure 1).

Periodontal disease is currently the 12th most prevalent condition worldwide. From 1990 to 2019, severe periodontitis cases accounted for 67.9% of the global population growth, which amounted to 1.1 billion cases in 2019 [5]. In addition, 90% of the global population has experienced some form of periodontal disease [6]. A recent literature review described the connection between periodontal disease and several health conditions, including diabetes, cardiovascular diseases, respiratory diseases, adverse pregnancy outcomes, Alzheimer’s disease, rheumatoid arthritis, and chronic kidney disease, highlighting the importance of addressing periodontal disease as a public health concern [7].

Periodontal disease treatment consists of different approaches, depending on the stage of the disease, based on supporting tissue involvement and alveolar bone loss. Non-surgical treatment involves scaling and root planing, a mechanical procedure to remove dental biofilm and calculus, carried out at sites where periodontal probing depths are equal to or greater than 5 mm [8]. Even after undergoing the periodontal pocket cleaning procedure, some subgingival biofilm may remain in the pocket, leading to a persistent chronic inflammatory response that can adversely affect the success of periodontal therapy [9]. Thus, additional therapies are essential to improve the outcomes of subgingival instrumentation alone. In this context, chlorhexidine and antibiotic therapy are adjunctive treatments.

Nevertheless, it is essential to mention that, although chlorhexidine is an excellent antimicrobial that is considered the gold standard chemical dental biofilm control agent, chlorhexidine mouthwash at 0.12% is not practical in preventing the development of gingivitis, in the presence of an established oral biofilm. Moreover, over 21 days of usage, adverse effects including taste alteration, numbness in the mouth and tongue, pain in the mouth and tongue, xerostomia [10,11], extrinsic tooth staining in long-term use [12,13], and development of antimicrobial resistance are associated with its usage. Furthermore, antibiotics, e.g., tetracycline, azithromycin, amoxicillin, and metronidazole, are an adjunctive therapy for periodontitis [14,15,16]. However, multidrug-resistant bacteria are a significant concern [17].

Areas with persistently deep periodontal probing depths indicate surgical periodontal therapy, reducing infrabony or vertical osseous defects using osteotomy and osteoplasty [18]. After that, the gingival tissue may be positioned apically at the new height of the alveolar crest; therefore, post-surgically, resective periodontal surgery may result in attachment loss in neighboring sites [19]; dentinal hypersensitivities from the exposed root surfaces [20]; and loss of interproximal papilla, resulting in chronic food impaction, aesthetic concerns, and phonetic change [21].

There are new tendencies in periodontal therapy such as adjunctive antimicrobial photodynamic therapy, probiotics, prebiotics/symbiotics, statins, pro-resolving mediators, omega-6 and -3, ozone, and epigenetic therapy. However, there currently needs to be more solid evidence to recommend them for daily practice [9]. In this context, natural compounds could be adjunctive, since plants and their phytochemicals have shown therapeutic properties. Herbal medications are suitable substitutes for synthetic medicines in preventing and treating periodontal disease, due to their considerable natural action, broader biological activity, substantial safeness, and lower price [22,23,24], in addition to their anti-inflammatory, antibacterial, and antioxidant effects [25].

**Figure 2:**
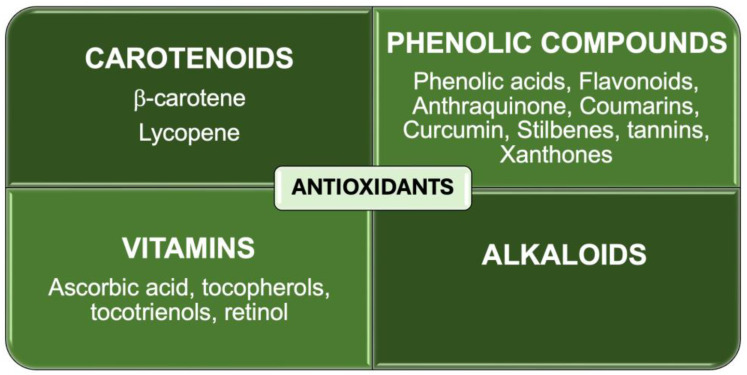
Nonenzymatic antioxidant types.

Antioxidants, both enzymatic and nonenzymatic, can counteract oxidative stress and prevent the progression of periodontal disease. Other pharmacological effects include antimicrobial effects, immune modulation, and anti-inflammatory effects [26]. Figure 2 shows a square diagram classifying nonenzymatic antioxidant types.

Therefore, this paper presents a patent mining related to antioxidant phytochemicals in the technological developments working to prevent and treat periodontal disease. Additionally, it explores the phytochemicals’ antioxidant properties in inhibiting oxidative stress pathways to prevent and treat periodontal disease.

### 1.1. Pathophysiology of Periodontal Disease

One key etiologic factor of periodontal disease is the dysbiotic biofilm formed by periodontal pathogens, e.g., *Porphyromonas gingivalis*, *Aggregatibacter actinomycetemcomitans*, *Tannerella forsythia*, *Eikenella corrodens*, and *Fusobacterium nucleatum*, which elicit an immune response to protect the host that controversially destroys supportive periodontal tissue [27]. 

Approximately 700 species of bacteria comprise the oral microbiome [28], a unique, organized, and diverse ecosystem living in homeostasis [29]. Planktonic bacteria such as *Streptococci* and *Neisseria* attach to a layer of macromolecules, called the acquired pellicle, in the oral cavity [30]. This attachment occurs due to weak bonds such as Lifshitz–van der Waals, Lewis acid–base, electrostatic interactions, and specialized appendages called fimbriae or pili, forming a dental biofilm [31,32].

Primary colonizing *Streptococci* and *Neisseria* bind to receptor sites of late colonizers such as *Fusobacterium nucleatum*, *Treponema denticola*, *Tannerella forsythia*, *Porphyromonas gingivalis*, *Prevotella intermedia*, or *Aggregatibacter actinomycetemcomitans* through coaggregation bridges, ultimately leading to the maturation of the dental biofilm [33]. The continued accumulation of supragingival and subgingival polymicrobial biofilm communities stimulates a persistent host immune response within the periodontium [34]. Chronic inflammation induces the development of a periodontal pocket that changes the redox and nutrient environment, increasing the diversity of the polymicrobial biofilm, developing a dysbiotic microflora [35], and activating an immune response.

During the host response against periodontopathic bacteria, the innate immunity cells, using Toll-like receptor 4 (TLR-4), recognize pathogen-associated molecular pattern (PAMP) Lipopolysaccharide (LPS) from *Porphyromonas gingivalis*, *Prevotella intermedia*, or *Aggregatibacter actinomycetemcomitans* [36]. The TLR4–LPS interaction transduces its signal to myeloid differentiation primary response gene 88 (MyD88) to downstream signaling molecules for inflammation. TLR4/MyD88 activates the Nuclear factor κ-light-chain-enhancer of activated B cells (NFκB), resulting in inflammatory mediators, such as interleukins (IL-1, IL-6, and IL-8), lytic enzymes, matrix metalloproteinases (MMPs) production, and the activation of osteoclasts. Moreover, LPS increases the osteoblastic expression of the receptor activator of the NF-kB ligand (RANKL), prostaglandin E2 (PGE2), and tumor necrosis factor-alfa (TNF-α), each known to induce osteoclastic activity, viability, and differentiation. It has been shown that RANKL is upregulated, whereas osteoprotegerin (OPG) is downregulated in periodontitis, compared to periodontal health, resulting in an increased RANKL/OPG ratio, leading to the destruction of the connective tissue attachment and alveolar bone loss and, consequently, the migration of the junctional epithelium apically [37,38,39,40,41]. IL-17 is also related to the pathogenesis of periodontitis. IL-17 and a dysbiotic microbiome might promote each other, enhancing microbiome pathogenicity and mucosal immunopathology [42]. 

Therefore, it is well established that the dysregulation or dysfunction of the immune response results in chronic and destructive inflammation [43]. On the molecular level, a non-specific inflammatory response releases reactive oxygen species (ROS), a subset of free radicals that contain oxygen, to protect the host against bacterial challenge. However, neutrophils in periodontal disease release excessive and prolonged ROS [44], due to a dysbiotic and persistent oral biofilm, leading to oxidative stress and tissue destruction.

### 1.2. The Role of Oxidative Stress in Periodontal Disease

Increasing evidence has shown that oxidative stress plays an essential role in the pathogenesis of various types of chronic inflammation, including periodontitis [45]. Neutrophils are the predominant sources of ROS [46], playing a leading protective role against invading bacteria through their antimicrobial mediators, phagocytosis, degradative enzymes such MMPs, and the production of reactive oxygen species with antimicrobial properties [47]. However, the ROS associated with chronic inflammation activate inflammasomes, resulting in the overproduction of ROS that destroy bacteria, leading to extensive tissue damage [43,44]. Therefore, protecting periodontal tissues or cells from oxidative stress can reduce periodontal tissue loss [48].

Excessive reactive oxygen species can upregulate signaling pathways that lead to the aggravation of periodontal inflammation. First, the nuclear factor-κB (NF-κB) signaling pathway is activated, which causes the expression of pro-inflammatory cytokines, chemokines, and MMPs. This process also activates the NOD-like receptor protein 3 (NLRP3) inflammasome, which is a complex of multiple proteins in the cytoplasm. It consists of NLRP3, ASC (apoptosis-associated speck-like protein, containing a CARD), and pro-caspase-1 [49]. According to studies, the NLRP3 inflammasome induces the secretion of pro-inflammatory cytokines IL-1β and IL-18 through the cleavage caspase-1. This process inhibits the migration of periodontal membrane fibroblasts and causes damage to the periodontal ligament. Ultimately, it leads to alveolar bone destruction [50]. Second, the RANKL signaling pathway is activated in a long-term inflammatory microenvironment, inducing osteoclastic activity and leading to alveolar bone destruction [51]. Third, continuously generated ROS not only inhibit human gingival fibroblast viability, but also cause apoptosis through a mitochondrial stress-mediated pathway [52]. In addition, nuclear factor erythroid 2-related factor 2 (Nrf2) is an essential antioxidant transcription factor. When Nrf2 is dissociated from Keap1 under oxidative stress, it translocates to the nucleus, binds to the antioxidant-responsive element (ARE), and upregulates endogenous antioxidants, providing protective effects. These effects include reducing inflammatory signaling pathways and oxidative damage in periodontal tissue [53].

**Figure 3:**
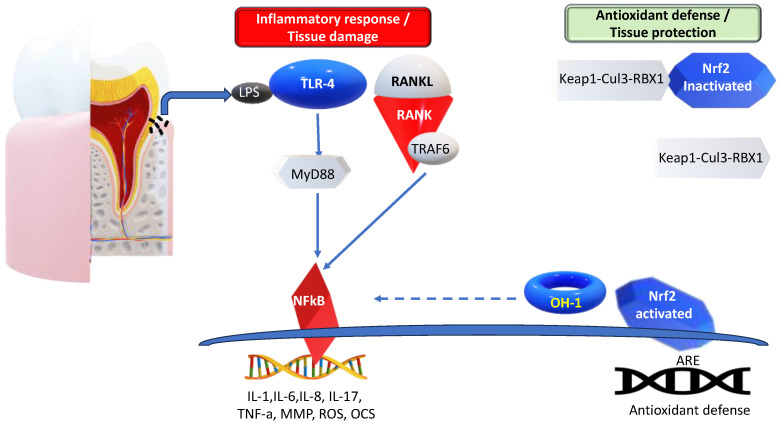
Molecular mechanisms of periodontal disease. LPS-TLR4/MyD88 activates NFkB, resulting in inflammatory mediators (IL-1, IL-6, IL-8, IL-17, and TNF-a), the activation of osteoclasts, MMPs, and ROS production. Moreover, LPS increases the osteoblastic expression of RANKL, to induce osteoclastic activity. Nrf2 is released from the Keap1-Cul3-RBX1 complex. It translocates into the nucleus and binds to the antioxidant response elements (AREs), regulating the expression of antioxidant defense. Arrows indicate activation, while dotted arrows show inhibition. LPS—lipopolysaccharide; TLR4—Toll-like receptor 4; MyD88—myeloid differentiation primary response gene 88; TNF-α—tumor necrosis factor-alfa; MMP—matrix metalloproteinase; ROS—reactive oxygen species; NFkB—nuclear factor κ-light-chain-enhancer of activated B cells; IL—interleukin; RANKL—receptor activator of NF-kB; Nrf2—nuclear factor erythroid 2-related factor 2.

Taken together, the activation of various pathways, namely the NF-κB and RANKL signaling pathways, as well as the JNK kinases and NLRP3 inflammasomes, contribute significantly to the destruction of periodontal tissue during chronic inflammatory oxidative stress. Moreover, the KEAP1-NRF2 stress response pathway plays an essential role in periodontal disease progression (Figure 3).

In order to counteract oxidant toxic effects, cells have developed antioxidant systems, including superoxide dismutases (SOD1–3), catalase (CAT), glutathione peroxidases (GPx1–8), thioredoxins (Trx1–2), and peroxiredoxins (Prx1–6), as well as nonenzymatic small molecules, for example, glutathione (GSH), α-tocopherol, and ascorbate [54,55]. An imbalance between the production of ROS and antioxidant defenses plays an essential role in the etiopathogenesis of periodontitis [56]. Therefore, a periodontal treatment capable of inducing antioxidant mechanisms to protect periodontal tissues is necessary.

### 1.3. Clinical Evidence for Using Antioxidants in the Treatment of Periodontal Disease

Periodontal disease causes inflammation and infection in the supporting structures of the teeth; therefore, antioxidants have been studied for their potential role in managing periodontal disease, due to their ability to counteract oxidative stress, which contributes significantly to the disease’s progression. The scientific literature describes clinical studies investigating the use of antioxidants in treating periodontal disease.

A study was conducted on 160 patients with periodontitis, randomly divided into four groups. These groups were orally administered either a placebo, a high dose (500 mg/d), a middle dose (250 mg/d), or a low dose (125 mg/d) of resveratrol, during an 8-week treatment. The results showed that the group receiving 500 mg/d of resveratrol significantly improved their periodontal health, specifically in clinical attachment level, bleeding index, oral hygiene index-simplified, and probing pocket depth, compared to the placebo group. Additionally, no adverse events were reported, concluding that resveratrol may be a safe and effective treatment for patients with periodontitis [57].

Locally administered drugs can achieve a high concentration in the periodontal pocket, reaching 100-fold higher therapeutic doses of the agent in the subgingival areas than systemic antimicrobial therapy. In addition, this method can improve clinical parameters without inducing systemic side effects [58]. In this context, a clinical trial was conducted on 30 patients divided equally into the Control Group and the Test Group. The Control Group received scaling and root planing treatment alone. In contrast, the Test Group received scaling and root planing treatment and a locally delivered gel containing 5% tea tree (*Melaleuca alternifolia*) oil (TTO). The results showed that the Test Group experienced a significant reduction in MMP-8, correlated with a reduction in clinical parameters related to disease severity. The study concluded that the single application of locally delivered antioxidants provides better therapeutic effects than scaling and root planing alone [59].

A randomized clinical trial compared the effects of ascorbic acid/platelet-rich fibrin and platelet-rich fibrin with surgical procedures, on patients with stage III grade periodontitis. The study examined the impact on clinical attachment level, probing pocket depth, gingival recession depth, full-mouth bleeding, plaque scores, radiographic defect bone density, and radiographic linear defect depth in intra-osseous defects. Both interventions significantly improved clinical and radiographic outcomes six months after surgery. Combining ascorbic acid with platelet-rich fibrin resulted in an additional significant improvement in gingival recession and radiographic defect fill, highlighting the positive impact of antioxidants in treating periodontitis. [60].

The Swedish national guidelines for adult dental care [61] recommend two evidence-based approaches for the initial nonsurgical treatment of patients with periodontitis. The first approach is focused on patient education, followed by a full-mouth ultrasonic instrumentation in one session. The second approach is a conventional nonsurgical therapy involving patient education and mechanical instrumentation (scaling and root planing), usually performed over several sessions. A recent study has shown that using a solution containing (-)-epigallocatechin gallate at a concentration of 5 mg/mL, as a water supply for ultrasonic scaling and root planing, can greatly improve the procedure’s effectiveness. Additionally, using this solution as an irrigant, instead of water, can also reduce the bleeding index. [62].

Studies conducted in the clinical setting indicate that antioxidants can help manage periodontal disease by reducing inflammation, improving gum health, and promoting tissue repair. However, more research is required to comprehend these antioxidants’ optimal dosages and mechanisms of action in periodontal therapy.

## 2. Materials and Methods

The following work was conducted by researching the Orbit Intelligence software licensed version v2.0.0 patent database documents. First, the keywords “PERIODONTAL” and “ANTIOXIDANT” were entered in the search field for the title, abstract, and claims sections. This search aimed to find patent documents related to the connection between antioxidant compounds and periodontal disease. The syntax used in the search was ((PERIODONTAL)/TI/AB/CLMS AND (ANTIOXIDANT)/TI/AB/CLMS)), where “TI” refers to the title field, “AB” refers to the abstract field, and “CLMS” refers to the claims field of the Orbit database. The results showed that the most cited compounds were lycopene, resveratrol, quercetin, curcumin, melatonin, green tea, and vitamin C.

Second, aiming to retrieve patent documents related to the association of the compounds (lycopene, resveratrol, quercetin, curcumin, melatonin, green tea, and vitamin C) mentioned with periodontal disease, the syntax ((name of the compound)/TI/AB/CLMS AND (ANTIOXIDANT)/TI/AB/CLMS)) was used, for example ((LYCOPENE)/TI/AB/CLMS AND (ANTIOXIDANT)/TI/AB/CLMS)).

Alive patents filed over the last 20 years were included in the second step of the study and the survey was carried out on 15 January 2024, according to the following exclusion criteria: (1) exclusion of the same and unrelated patents; (2) exclusion of patents related to synthetic antioxidants; and (3) exclusion of patents related to devices used for administration and methods to extract bioactive compounds.

This study focused on patent families, which refer to patents related to the same invention developed by one or more inventors and patented in multiple countries. To analyze the data, we utilized the graphical representations provided by the database and electronic spreadsheets to organize the information obtained.

The patent families found were analyzed, focusing on the world distribution of technologies and potential markets, the annual evolution of the first application, technological domains, applicants, and a detailed analysis of each technology.

The next step was to evaluate the market. The intellectual property market for natural antioxidants extracted from medicinal plants to treat or prevent periodontal disease was evaluated using only granted patent documents. For this purpose, the syntax (((((PERIODONTAL)/TI/AB/CLMS AND (ANTIOXIDANT)/TI/AB/CLMS)) AND STATE/ACT=ALIVE) AND STATUS/ACT=GRANTED) was used.

The Intellectual Property (IP) investment (the sum of the costs of patent families on the topic) was calculated using the Questel orbit platform algorithm. This algorithm estimates the cost of each procedure in 2018, 2019, and 2020, according to the patents still alive in each patent family. The value is set based on a “known” market of comparable size. This study considered the IP market size of graphene batteries, which is a small market. The IP Market size was classified as Small (<USD 10M), Medium (between USD 10 and 100M), or Big (>USD 100M).

The market strategy was calculated using the Questel orbit platform algorithm. The market strategy of an invention corresponds to the sum of the Gross Domestic Product (GDP) of the countries protected by the patent family. GDP is a monetary measure of the market value. Data were normalized to give a score of 1 to an invention that only covers the US with a granted patent. The qualification of the Market Strategy was classified as Local (<0.6), Main market (>0.6 and <0.9), or Global (>0.9).

Finally, we have presented the perspectives for the analyzed sector, considering the information compiled.

## 3. Results and Discussion

### 3.1. Overview of Antioxidant-Related Patents in Periodontal Diseases

**Figure 4:**
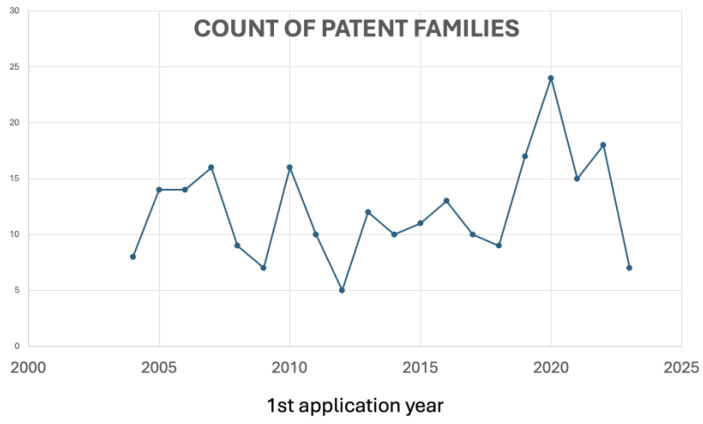
Evolution of applications over time of antioxidant-related patents in periodontal disease.

Regarding the patent literature, a total of 322 patent documents has shown that, over the last 20 years, patent publications related to the use of antioxidants to treat or prevent periodontal disease exhibited an increased number of documents between 2019 and 2020 (Figure 4), in line with the increasing number of scientific paper publications between 2019 and 2022, related to the growing interest in researching natural antioxidants with scientific and technological purposes. 

**Figure 5:**
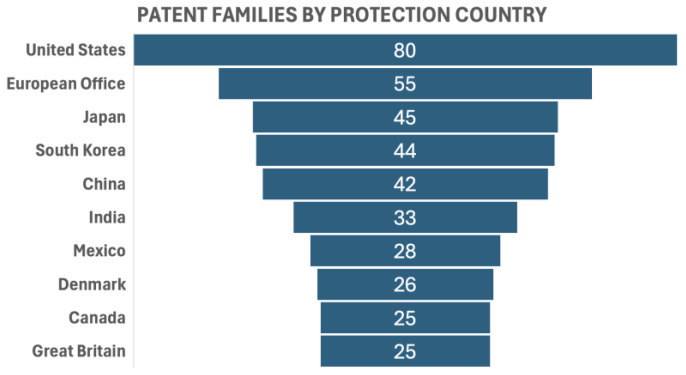
Patent families by protection country.

The top ten countries in terms of the number of family patents produced were the United States (80 family patent documents), the European Office (55), Japan (45), South Korea (44), China (42), India (33), Mexico (28), Denmark (26), Canada (25), and Great Britain (25). A patent family is a collection of patent applications covering the same or similar technical content [63] (Figure 5).

The most cited compounds were vitamin C, green tea, quercetin, melatonin, lycopene, resveratrol, and curcumin. The analysis of studies conducted in the patent documents highlighted these phytochemicals as promising compounds to be considered for developing oral healthcare products. 

### 3.2. Vitamin C-Related Patents in Periodontal Diseases

Regarding patent documents, 66 were analyzed after applying exclusion criteria from 88 retrieved vitamin C-related patents. The top three countries in terms of the number of patents produced are the US, South Korea, and China. These countries are witnessing a surge in vitamin demand, attributed to consumer awareness of the importance of a well-balanced diet and a focus on preventive healthcare. In line with this goal, vitamin C is a promising compound. Also known as ascorbic acid, vitamin C is a water-soluble vitamin with well-established antioxidant properties [64]. It participates in collagen synthesis [65], wound healing [66], and immune function [67]. Vitamin C is a potent antioxidant and a scavenger of ROS. Its biological relevance is associated with the recovery of α-tocopherol and the prevention of endothelial dysfunction [68].

The potential use of vitamin C as an antioxidant in treating periodontal disease is grounded in its ability to counteract oxidative stress and support overall oral health. An in vitro study evaluated the effect of vitamin C on the primary culture of human periodontal ligament stem cells exposed to *P. gingivalis* lipopolysaccharide. Vitamin C downregulated the inflammatory pathway and ROS generation and modulated the miR-210 level, a crucial factor in osteogenic and angiogenic processes [69,70]. Immunostaining in the gingival tissue of rats with experimentally induced periodontitis and diabetes [71] showed that vitamin C reduces the following: (a) tissue advanced glycation end products (AGEs), involved in hyperinflammatory response, oxidative stress, and bone destruction [67] and (b) matrix metalloproteinases-8, responsible for the degradation of type 1 collagen in supportive periodontal tissue [71], showing immunomodulatory activity, tissue damage protection, as well as tissue repair promotion, reaffirming the therapeutic potential of vitamin C for periodontal disease treatment. However, a randomized controlled trial did not prove that a supplemental dose of vitamin C, along with nonsurgical periodontal therapy, in chronic periodontitis patients has benefits [72], highlighting the need for further studies.

Most daily vitamin C intake comes from fruits and vegetables [64]. The US vitamin C market is projected to expand at a compound annual growth rate (CAGR) of 5.4% between 2023 and 2027 [73], explaining the US position as a vitamin C-related patent application leader. Several pharmaceutical companies are using innovative technologies in manufacturing oral care products using vitamin C; Spoke Sciences is the leader among them [74]. The patent EP3445356 belongs to Spoke Sciences and relates to a vitamin C herbal composition. This composition is designed for oral delivery and has multiple benefits, such as enhanced bioavailability, absorption, and a faster onset of action. It also provides higher peak concentrations, a faster time to reach them, and increased subjective and objective therapeutic efficacy. The herbal composition’s formulation includes active components such as vitamin C, rutin, quercitrin, curcumin, resveratrol, limonene, and linalool; N-acylated fatty amino acids (linear, branched, cyclic, bicyclic, or aromatic); and extracts from plants such as *Calophyllum brasiliense*, *Salix alba*, *Calendula officinalis*, *Camellia sinensis*, *Melissa officinalis*, *Curcuma longa*, *Vitis* spp., *Capsicum* spp., *Vinca* spp., *Citrus* spp., *Lavandula* spp., *Mentha* spp., *Cannabis sativa*, and *Acer* spp., among others.

The oral care industry is highly competitive; companies always look for new ingredients or formulations to make their products stand out. Adding vitamin C to a formulation for developing innovative oral care products could provide a unique selling point and attract consumers interested in natural, safe, environmentally friendly, and vitamin-enriched products.

The oral hygiene composition is formulated as a dentifrice, chewing gum, orally disintegrating film, mouthwash, mouth spray, or mouth massage cream. Mouthwashes were prominent among the developed technologies. The patent KR102372384, entitled “Oral hygiene composition for preventing or alleviating periodontal disease and halitosis”, comprises collagen, *Ginkgo biloba*, vitamin C, tocopherol, lysozyme chloride, a zinc compound, and *Allium sativum* extract as active ingredients and is effective in suppressing periodontal diseases.

In a patent document entitled “Liquid toothpaste and preparation method thereof” (CN104666185), a mouthwash was described as a tooth–liquid paste. The toothpaste is made from a variety of ingredients including xylitol, vitamin C, cetylpyridinium chloride, sodium fluoride, natural betaine, sodium phytate, traditional Chinese medicine extracting solution, lauryl sodium sulfate, sodium lauroyl sarcosine, glycerol, sorbitol, silica, essence, a freshener, antiseptic, a sweetening agent, edible gum, and water. The toothpaste is liquid, allowing active ingredients to easily reach all mouth surfaces and penetrate gaps in the teeth. The inventors claim that liquid toothpaste is effective in inhibiting bacterial growth, as well as reducing gingival bleeding and swelling.

Dental implants are the gold-standard treatment for tooth loss caused by periodontal disease. However, inflammation and bone loss in dental implants are common biological complications. The patent US20230270761, entitled “Immunomodulatory, oral microbiome altering and tissue regenerative oral care compositions and methods of use in the prevention and treatment of periodontal and peri-implant diseases”, describes the use of a therapeutic hydrogel in nonsurgical techniques, to promote the healing and regeneration of periodontal tissues and to treat peri-implant diseases, comprising ingredients as follows: water, glycerin, *Aloe vera* powder, tetrahydrocurcumionoids, xylitol, glycerin, vitamin C, peppermint oil, spearmint oil, Chinese Star Anise extract, and myrrh oil.

A vitamin C deficiency is associated with scurvy, characterized by bleeding gums [75]. Adequate vitamin C levels may help to reduce the likelihood of bleeding gums associated with gingivitis. This property was explored in the patent US10022412, entitled “Composition for preventing or alleviating periodontal diseases, containing, as active ingredient, mangosteen extract or α- or γ-mangosteen”. The invention has antibacterial effects against periodontopathogens *Prevotella intermedia* and *Fusbacterium nucleatum* and anti-inflammatory activity, comprising a daily dosage of 7.5 to 9.0 mg of alpha-mangosteen together with propolis, lysozyme chloride, soybean unsaponifiable matter, vitamin C, xylitol, and vitamin E.

**Figure 6:**
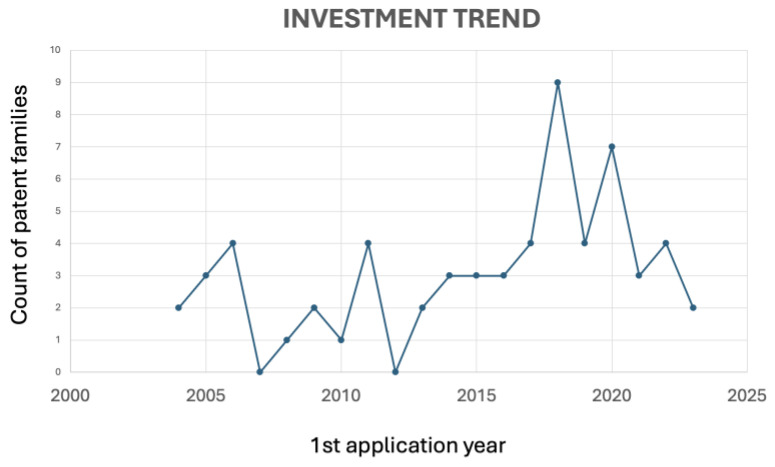
Evolution of applications over time of vitamin C-related patents.

Figure 6 illustrates the evolution of applications over time, indicating the dynamics of the inventiveness of the patents studied. There will always be a gap in current patent information, due to the 18 month delay between filing an application and its publication, which explains the numbers in 2023 and 2024. 

The first patent (KR100687037) was granted to Hongkeun, Ji Hong Keun, Choi, and Jeong Sik. It described the composition of a nanocarrier for oral care, containing vitamin C, recithin, ethanol, capric/caprylic triglyceride, balsam extract, tocopherol acetate, licorice extract, cetyl phosphate, BHT (butyl hydroxy toluene), and purified water. The present invention aims to improve the absorption of oil-soluble licorice extracts in oral mucosa. Due to their poor solubility, these extracts have low absorbency on skin or gums.

The nanotechnology structures range from 1 to 100 nm, a submicroscopic size, and provide practical implementations in a wide range of fields [76], including engineering, drug delivery, nanomedicine, environmental indemnification, catalysis, and target diseases [77]. Therefore, medicines linked with nanotechnology have their efficiency and bioavailability increased [78]. In periodontal disease, bacteria embedded in the dental biofilms are protected from environmental stress stimuli and show reduced responses to antibiotics, making it difficult to eradicate the infection [79]; therefore, nanocarriers can target specific sites in the oral environment, such as inflamed periodontal pockets, reducing the potential side effects and maximizing the therapy.

There is a growing trend towards more natural and sustainable products; using vitamin C in oral care products could align with this trend. Moreover, funding research into the potential benefits of vitamin C in oral care products could lead to new patents and products, improve public health, foster innovation, and support economic growth. On the other hand, it is important to highlight that, although vitamin C is generally safe, excessive doses can lead to adverse effects [80] and that its clinical effectiveness in periodontal disease is not yet well established. Therefore, the treatment should be assessed on a case-by-case basis, according to patients’ health conditions and needs. 

### 3.3. Green Tea-Related Patents in Periodontal Diseases

**Figure 7:**
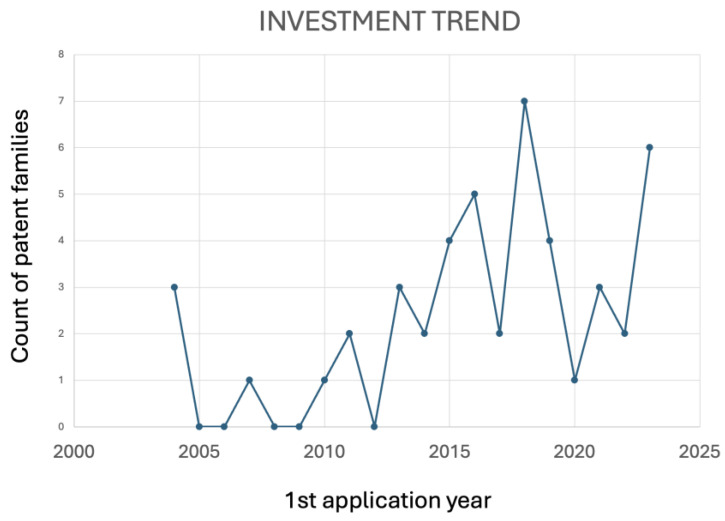
Evolution of applications of green tea-related patents over time.

**Figure 8:**
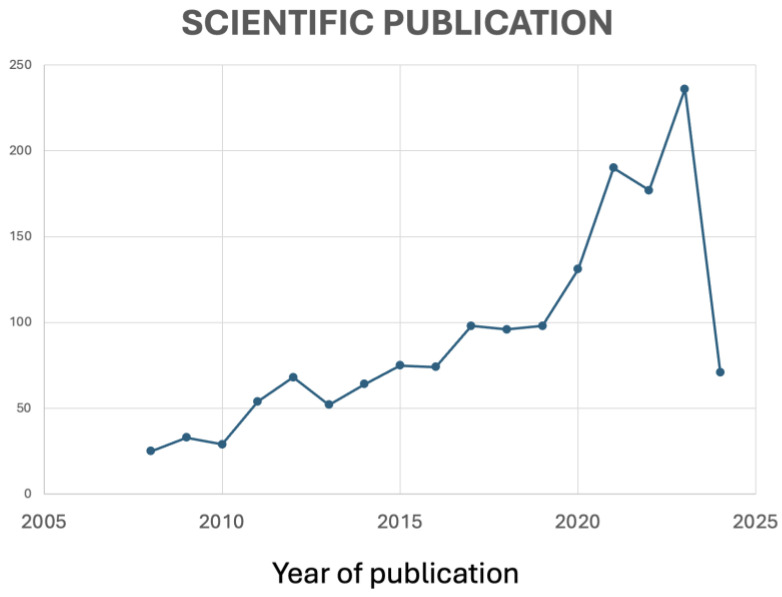
Evolution of published papers over time.

Green tea, derived from the *Camellia sinensis* plant, is known for its rich content of polyphenols, particularly catechins [81]. Its antioxidant properties may contribute to periodontal health. Green tea catechins, especially epigallocatechin gallate, exhibit antioxidant [82] and anti-inflammatory effects, in addition to supporting bone health [83]. Green tea was the second most cited compound in the patent literature. According to Figure 7 and Figure 8, it seems that research projects conducted by universities on using green tea in oral care have led to new technological developments in this field.

Academic research and technological development are interdependent, each driving progress in the other. This interaction is vital for promoting innovation and addressing societal challenges. However, many universities prioritize academic research and education, over commercialization. Additionally, introducing new technologies to the market often involves navigating complex regulatory frameworks and addressing ethical concerns, which academic researchers may need more expertise or resources to handle. Although there are challenges, Figure 7 and Figure 8 seem to demonstrate that innovative ideas and solutions produced by universities can be applied to technological advancements. For example, in 2018, the peak observed in Figure 7 correlates with the exponential growth of scientific publications between 2015 and 2023 in Figure 8.

**Figure 9:**
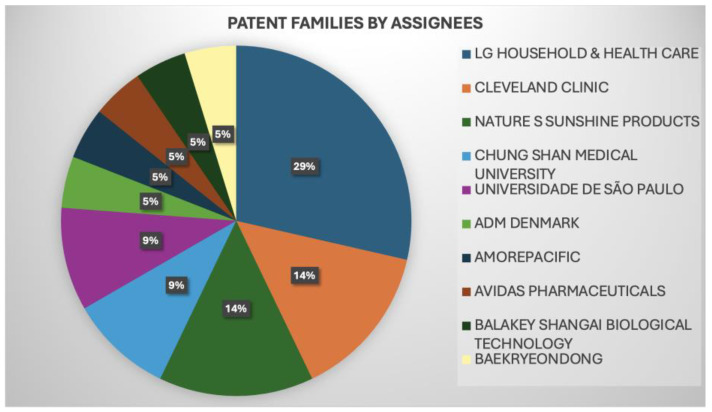
The size of the applicants’ portfolios in the green tea-related patents analyzed.

Similarly, Figure 9 showcases the leading players and their key inventions, referred to as green tea-related patents, that have undergone litigation, opposition, licensing, or have been cited in a standard. Patents that have successfully survived litigation or opposition are recognized as solid patents. Figure 9 displays the top players in the industry (LG Household and Health care/Cleveland clinic/Nature S Sunshine Products) and in academia (Chung Shan Medical University/Universidade de São Paulo), showing that universities and industries have performed academic research and technological development, a positive indicator for scientific development. 

Green tea (*Camellia sinensis*) is consumed because of its therapeutic effects. It contains polyphenolic compounds, especially flavanols called catechins, such as epigallocatechin gallate (EGCG), a potent antioxidant [74]. EGCG increases the expression of bone morphogenetic protein-2, runt-related transcription factor 2, alkaline phosphatase, osteonectin, and osteocalcin; increased alkaline phosphatase activity improves mineralization [83]. The ability of a mucoadhesive gingival patch loaded with EGCG (GP-ECGC) on a periodontitis model induced by *Porphyromonas gingivalis* was investigated [84]. The results showed that the GP-EGCG treatment for 3 days, up until 21 days, consistently increased the IL-10 expression in periodontitis. Conversely, GP-EGCG treatment lowered the IL-6 expression after 7, 14, and 21 days. 

Regarding green tea-related patents, oral patches are a crucial study area for developing technological products. Oral patches are an additional method to improve the standard mechanical treatment of periodontitis, which involves scaling and root planing. This patch is known for its ability to hold the medicine for a prolonged period at the application site, making it an effective technique. For instance, the patent document KR102309837 describes an innovative patch that can be attached to a tooth or a peripheral portion. This patch can be easily removed by brushing, without removing the support layer that holds the medicinal layer containing at least one active ingredient like Sanguinarine or Triclosan. It may also contain herbal extracts such as pumpkin, *Centella asiatica*, chamomile, *Latania molasses*, barnyardgrass, kiln, green tea, licorice, barnyardgrass, or gold hiding.

Companies are also exploring the possibility of developing oral care products such as toothpaste, as described in the following patent documents: KR101955468, “Toothpaste composite of power type”; WO201534252, “Preparing method for toothpaste composition comprising natural extracts”; KR102622954, “Toothpaste containing medicinal herb extract and propolis”; and KR102162661, “Toothpaste composition for improving or preventing gingivitis and periodontitis”, providing a more holistic approach to oral health. However, it is essential to note that more research is required to fully comprehend the extent of green tea’s benefits and how it can be effectively integrated into oral care products.

### 3.4. Quercetin-Related Patents in Periodontal Diseases

Quercetin is a typical flavonoid compound in fruits and vegetables, such as apples, potatoes, tomatoes, and onions [85], with numerous pharmacological properties already being described [86,87,88,89]. In total, 27 patent documents were analyzed after applying exclusion criteria from 71 retrieved quercetin-related patents. The top three countries in terms of the number of patents produced are China, Japan, and the US. In 2019 and 2023, the number of patent filings was highest. Over time, the development of the patent system in China has seen a shift towards better protection of intellectual property rights and alignment with international standards. China’s focus on innovation and technological development has also contributed to the growth of its patent system, which now ranks as one of the top patent-filing jurisdictions globally.

An analysis of the most used concepts in the quercetin-related patents found that quercetin’s anti-inflammatory activity was the most frequently referenced. In fact, quercetin inhibits the M1 inflammatory macrophage, suppressing the damage in periodontal tissue and promoting M2 polarization for regenerating the surrounding tissues in the reparative phase of periodontal disease [86]. In addition to its anti-inflammatory action, quercetin is a potent antioxidant compound. Quercetin enhanced the expression of antioxidant enzyme-related genes, including HO-1, GPx3, and CAT, and elevated SOD activity in an in vitro study, in addition to activating Nrf2 in periodontal ligament cells [89]. Nrf2 is a transcription factor related to antioxidant response, which regulates the expression of antioxidant genes during oxidative stress [87]. The expression of Nrf2 is related to periodontal health tissues. Quercetin can increase the antioxidant capacity of periodontal ligament cells and reduce oxidative stress damage by activating the Nrf2 signaling pathway, which protects alveolar bone loss in periodontitis. In addition, it is assumed that Nrf2 increases the cellular HO-1 expression and inhibits the oxidative stress mediated by NFkB activation, blocking the degradation of IκBα [88].

Based on the great therapeutic potential of quercetin, different delivery systems and innovative formulations have been developed with this compound, aiming to improve its pharmacological properties and optimize its therapeutic effects in periodontal disease. Among them, the patent CN116726243, “Quercetin/polycaprolactone fiber electrospun membrane as well as preparation method and application thereof”, provides a biocompatible quercetin/polycaprolactone fiber electro-spun membrane, capable of inducing the osteogenic differentiation of stem cells and guiding periodontal tissue regeneration. The patent US20230270761, “Immunomodulatory, oral microbiome altering and tissue regenerative oral care compositions and methods of use in the prevention and treatment of periodontal and peri-implant diseases”, describes a therapeutic hydrogel in nonsurgical techniques, to promote the healing and regeneration of tissues whose structure and functions have been diminished due to chronic inflammatory disorders, including periodontal disease.

Products that induce osteogenic differentiation and wound repair in periodontal treatment hold promise for improving the outcomes of various therapeutic approaches and enhancing the overall management of periodontal disease, which results in bone loss around the teeth. Inducing osteogenic differentiation can restore the supportive structures of the teeth, which is especially useful in cases where advanced periodontal disease has caused significant bone resorption. By improving wound repair mechanisms, the technology can speed up the healing process, reduce inflammation, and promote the regeneration of periodontal ligaments and connective tissues. On the other hand, while some studies suggest that quercetin may be beneficial in treating periodontal disease, further research is needed to understand its effectiveness fully. The patented technology can be used alongside traditional periodontal treatments, such as scaling and root planing, to promote tissue regeneration and improve long-term clinical outcomes. Table 1 presents quercetin-related patents in periodontal disease.

### 3.5. Melatonin-Related Patents in Periodontal Diseases

A granted patent (US8658139) described one innovative compound for the control of periodontal disease—melatonin. Although melatonin is primarily known for regulating the sleep–wake cycle [90], its antioxidant properties have pointed to this phytochemical as a possible application in treating periodontal disease. The patent explored the topical application of melatonin in gels or mouthwashes. This targeted delivery may enhance the local effects of melatonin in the oral cavity, directly addressing periodontal tissues. Additional features of this invention include the prevention and treatment of aphthous ulcers, oral mucositis, periodontal disease, perioral dermatitis, halitosis, oral candida, chapped lips, oral plaque, xerostomia, and cold sores.

Melatonin (N-acetyl-5-methoxytriptamine) is an indolamine, initially discovered in 1958 in extracts from the bovine pineal gland [91]. For over 30 years, it was assumed that melatonin was exclusively produced in the pineal glands of animals; however, nowadays, it is known that melatonin is also produced by several organisms, including plants [92]. Melatonin interacts with melatonin receptor 1 (MT1) and melatonin receptor 2 (MT2) in cells, mainly exerting antioxidant effects [93]. Moreover, melatonin improved periodontal parameters, including bleeding on probing; probing depth; clinical attachment level; decreased pro-inflammatory cytokines IL-1b, IL-6, and TNF-α [94]; and stimulated the proliferation of osteoblasts, promoting bone formation [95]. However, these results were not corroborated by the study conducted by Konečná and coworkers, analyzing the effect of exogenous melatonin in an animal model of periodontitis and in patients with periodontitis [96]. In rats, melatonin was administered in drinking water for two weeks. In the human study, patients were asked to rinse their mouths with a solution containing melatonin or a placebo every evening for two weeks. The results do not support the use of melatonin for the treatment of periodontitis, highlighting the need for additional studies.

Combining natural compounds with antibiotics can improve treatment outcomes and overcome some difficulties associated with antibiotic resistance and side effects. This combined approach can produce a synergistic effect that enhances the efficacy of the treatment. In line with this idea, the patent RO-133752 describes a topical treatment of periodontal lesions, based on the association between melatonin–hyaluronic acid/tetracycline and metronidazole, which is a biocompatible paste used in the topical treatment of slowly progressive chronic marginal periodontitis. According to the invention, the material consists of three parts by weight of tetracycline; three parts by weight of metronidazole; 0.18 parts by weight of melatonin; three parts by weight of hyaluronic acid, with an average molecular weight of 300kDa; and, for the remainder, up to 100 parts white petrolatum, the material being in the form of complex matrix, in which the active principles have a synergistic action in biological environments.

### 3.6. Other Compound-Related Patents in Periodontal Diseases

Other compounds, such as curcumin, lycopene, resveratrol, and plant extracts, were also described in patents, showing that phytochemicals with antioxidant, anti-inflammatory, and bone-protective effects are promising candidates for developing oral health products.

Curcumin is a polyphenol, mainly extracted from the rhizome of *Curcuma longa* that possesses potent anti-inflammatory and antioxidant effects [97], due to the downregulation of cyclooxygenase, lipoxygenase, inducible nitric oxide synthase, soluble vascular cell adhesion molecule −1, cytokines, and NF-κB [98]; furthermore, curcumin promotes the osteogenic differentiation of human periodontal ligament stem cells, enhancing the bone repair ability of these cells in vivo [99]. The potential of this compound as an adjuvant in the treatment of periodontal disease has been demonstrated. A two-time application of curcumin oral gel along with scaling and root planing at one-week intervals resulted in decreased bleeding on probing, probing depth, and a gain in clinical attachment level in both groups (test and control) from baseline to 45 days [100]. Oral administration of curcumin and rutin, single or combined, could reduce oxidative stress in the gingival tissue and blood and could enhance the antioxidant status in hyperglycemic periodontitis rats [101]. The authors showed that glutathione (GSH), GSH/glutathione disulfide (GSSC), and catalase activities significantly increased. Furthermore, levels of malondialdehyde (MDA), a compound derived from lipid peroxidation [102], significantly decreased compared to the placebo group, after administering rutin and curcumin. MDA is associated with the expression of pro-inflammatory genes and the activation of downstream inflammatory signaling pathways, including protein kinase-C, p38-MAPK, ERK1/2, and NF-κB [103].

Technological developments related to the use of curcumin to treat or prevent periodontal disease were found, as follows: (1) The patent CN116617208, “Application of composition in improving performance of senescent periodontal ligament stem cells”, discloses the application of a composition (curcumin and wogonin) in improving the performance of senescent periodontal ligament stem cells, promoting the osteogenic differentiation of periodontal ligament stem cells from older people; (2) The patent CN117180227, “ Curcumin-loaded manganese-doped hollow mesoporous silica nanoparticle and application thereof in periodontal bone tissue repair”, provides a curcumin-loaded, manganese-doped hollow mesoporous silica nanoparticle for bone tissue repair. According to the application, bone targeting is achieved by grafting alendronate on the surface of the silicon dioxide nanoparticles, manganese is doped on the nanoparticles using a chemical method, and curcumin is wrapped in the nanoparticles; and (3) The patent IN202041057119, “Injectable subgingival gel”, describes an antibacterial syringeable gel containing *Aloe vera*, nutmeg, and curcumin, delivered subgingivally into the periodontal pocket.

Another promising compound is resveratrol (3, 5, 4′-trihydroxystilbene), a polyphenol mainly found in *Polygonum cuspidatum* roots [104]. In addition to being a potent antioxidant compound, resveratrol is an anti-inflammatory agent, reducing TNF-α, IL-1β, IL-6, MMP, and COX-2, induced by NF-κB activation [105,106]. Pre-clinical and clinical studies have already demonstrated the therapeutic effects of resveratrol in periodontal disease. A ligature-induced periodontal disease model in mice evaluated resveratrol derivative-rich melinjo seed extract (MSE) activity at 0.001% (*w*/*w*). The morphometric analysis showed a lower bone loss in the MSE groups than the control group, in addition to lower oxidative stress and the inhibition of M-CSF/sRANKL, downregulating osteoclast activity, promoting the healing of periodontal bone loss, and modulating immune–inflammatory systems [107]. A total of 43 patients with chronic generalized periodontal disease were prescribed Resverazin, one capsule two times a day, a composition in which the main active ingredients are resveratrol (150 mg), black wine extract (100 mg), and grape seed extract (50 mg). Positive clinical outcomes include reducing the severity of symptomatic gingivitis and the depth of the periodontal pockets, showing that resveratrol is a promising drug to treat periodontal disease [108].

A patent document, known as “Resveratrol liposome as well as preparation method and application thereof” (CN115607511), explains how to prepare a resveratrol liposome from raw materials such as egg yolk lecithin, cholesterol, distearoyl phosphatidyl ethanolamine-polyethylene glycol 2000, and resveratrol. Liposomes are tiny, spherical-shaped vesicles made of lipids and are used as drug-delivery systems for small molecules, peptides, genes, and monoclonal antibodies. They have helped treat patients with various conditions, such as cardiovascular disease, neurodegenerative disease, diabetes, cancer, and inflammation [109]. According to the inventors, the invention CN115607511 has the following beneficial effects: (1) the resveratrol liposome has a slower release effect than a free drug; (2) M1-type macrophages can absorb the resveratrol liposome, effectively inhibiting their pro-inflammatory capacity, in addition to determining the polarization of the pro-inflammatory M1-type macrophages to anti-inflammatory M2-type macrophages; and (3) the resveratrol liposome inhibits the activation of NF-kB and NLRP3 inflammasomes, thereby inhibiting the expression of IL-6, IL-1 beta, IL-12, and TNF-alpha and promoting the expression of anti-inflammatory factors, including IL-10.

Another compound mentioned is lycopene, a natural antioxidant, exhibiting a physical quenching rate with singlet oxygen twice as high as beta-carotene and ten times higher than α-tocopherol. A clinical study has concluded that oral lycopene and green tea extract supplementation are positively associated with salivary uric acid (antioxidant) levels, which are essential in managing gingivitis [110].

The proliferative effects of lycopene on the human osteoblast cell line (CRL-11372) were analyzed using an in vitro wound healing model. Lycopene had proliferative effects on human osteoblasts, as well as a helpful approach in tissue engineering procedures and periodontitis treatment [111]. In fact, the effect of lycopene on osteoblasts and osteoclasts was presented in the patent EP2948005, a therapeutic composition with anti-inflammatory activity. It contains a heat-treated clear tomato concentrate (CTC), obtained by heating the CTC at 90 °C. The invention relates to a synergistic combination of the heat-treated tomato-derived concentrate with carotenoids such as lycopene, phytoene, phytofluene, beta-carotene, and lutein and/or their derivatives. Research shows that the composition has positive effects on bone health by reducing osteoclast differentiation and stimulating antioxidant response element signaling (ARE/Nrf2) in osteoblasts.

Alternatively, the patent literature has described the antioxidant, anti-gingivitis, and anti-inflammatory activities of medicinal plant extracts used to develop technologies for treating periodontal disease. Among the medicinal plants mentioned, the following stand out: *Zingiber ginger* and *Alpine officinarum* [112], *Pepper nigrum*, *Olea europae* [113], *Lycium barbarum* [114], red tea [115], *Acacia nilotica* (L.) Delile [116], *Aegiceras corniculatum* [117], *Calendula officinalis* [118], tea tree oil and *Aloe vera* [119], Triphala, Ayurvedic medicinal herbal formulations [120], *Ficus deltoidea* [121], *Arctium lappa*, *Zataria multiflora* and *Echinacea purpurea* [122], *Pistacia lentiscus* L. [123], *Curcuma longa* [124], *Nigella sativa* [125], and *Chrysopogon zizanioides* [126].

Other plant extracts have been mentioned in the patent literature. A document named “Method for alleviating gingivitis and periodontitis by antibiosis, antioxidation, anti-inflammation, suppression of periodontal bone loss, and regeneration of periodontal bone of complex of Moringa” (EP3603422) outlines a composition that can prevent and improve gingivitis and periodontitis. The active ingredient in this composition is derived from *Moringa* extract and *Eucommia* bark extract. It can effectively treat periodontitis by reducing inflammation in damaged gingival tissue, inhibiting alveolar bone loss, and promoting regeneration beyond just alleviating the symptoms of gingivitis and periodontitis.

Another example is the patent “Complex for the prophylaxis of oxidative stress in the oral cavity” (WO202120994). The invention relates to a complex of active ingredients for use in agents for teeth and oral cavity care, based on an extract of *Rosa canina*, a dry extract of *Magnolia officinalis*, and *Citrus clementina* essential oil. The purpose of the invention is to reduce oxidative stress in the oral cavity through prophylactic use.

The document, identified as “Nontoxic Dental Care Herbal Formulation for Preventing Dental Plaque and Gingivitis” (EP1641537), presented an oral care composition that included a synergistic herbal formulation. This formulation consisted of *Azadirachta indica* (2–5.5%), *Citrullus colocynthis* (0.5–2.5%), and an antioxidant derived from *Cucumis sativus* extract (0.1–0.4%).

The following is a formula that can be used to prevent and treat periodontal infections. It includes 0.2% to 12% by weight of an extract of *Solanum xanthocarpum*, 0.01% to 5% by weight of essential oil, 0.01% to 10% by weight of oral care active agents, 0.001% to 5% by weight of preservative, 0.01% to 5% by weight of flavoring agents, 0.01% to 15% by weight of sweetening agent, and 40% to 90% by weight of aqueous carrier. This formula has antibacterial, antioxidant, and anti-inflammatory effects, promoting overall oral health-related quality of life for the individual.

### 3.7. Targets of Antioxidant Phytochemicals in Periodontal Disease

**Figure 10:**
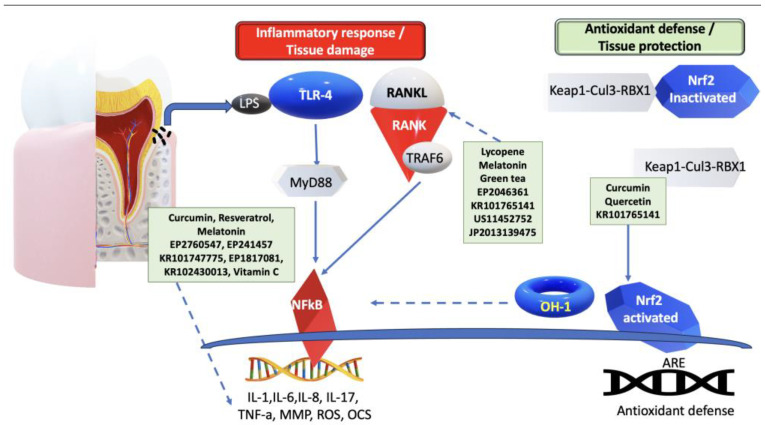
Antioxidant phytochemicals—application in the prevention and treatment of periodontal disease. Arrows indicate activation, while dotted arrows show inhibition. LPS—Lipopolysaccharide; TLR4—Toll-like receptor 4; MyD88—myeloid differentiation primary response gene 88; TNF-α—tumor necrosis factor-alfa; MMP—matrix metalloproteinase; ROS—reactive oxygen species; NF-kB—nuclear factor κ-light-chain-enhancer of activated B cells; IL—interleukin; RANKL—receptor activator of NF-kB ligand; Nrf2—nuclear factor red line 2 related factor 2; AREs—antioxidant response elements.

Oxidative stress is a critical factor in periodontal disease, harming periodontal tissues and worsening inflammation. Figure 10 displays some of the main antioxidant targets in periodontal disease and shows the possible applications of the technologies described.

As shown in Figure 10, while LPS-TLR4/MyD88 activation triggers the activation of NF-kB, leading to the production of inflammatory mediators such as IL-1, IL-6, IL-8, IL-17, as well as TNF-α, it also results in osteoclast activation; MMPs; ROS production; antioxidants such as curcumin, resveratrol, Melatonin, and Vitamin C; and innovative technological developments, as described in patent documents EP2760547, EP241457, KR101747775, EP1817081, and KR102430013, exhibiting anti-inflammatory effects and helping to control inflammation. Additionally, LPS amplifies the osteoblastic expression of RANKL, to promote osteoclastic activity. However, certain antioxidants such as lycopene, melatonin, green tea, and the latest technological advancements mentioned in patent documents EP2044361, KR101765141, US11452752, and JP2013139475 can effectively inhibit RANKL expression, thus controlling osteoclast activity and safeguarding alveolar bone resorption. Nrf2 is released from the Keap1-Cul3-RBX1 complex. It translocates into the nucleus, binds to AREs, and regulates antioxidant defense expression. Curcumin, quercetin, and the technological developments described in patent document KR101765141 could improve this process, protecting periodontal tissue.

In summary, combining natural antioxidants with conventional or surgical periodontal treatments can improve the overall management and outcomes of periodontal disease, by neutralizing harmful free radicals in gum tissues, supporting the healing process, and reducing tissue damage. The scientific literature and patent data have shown that combining scaling and root planing with antioxidants improves clinical attachment level, probing pocket depth, gingival recession depth, full-mouth bleeding, and plaque scores. Additionally, antioxidants can modulate immune function, reducing the progressive destruction of periodontal supportive tissues, mediated by an aberrant immune response against dysbiotic microbial communities. Antioxidants may have systemic health benefits beyond periodontal tissues, indirectly contributing to better periodontal health outcomes. Based on the findings, dentists can consider using antioxidants in clinical practice through the following methods: (1) administering resveratrol orally for an 8-week periodontal treatment, according to [57]; (2) utilizing antioxidants in periodontal pocket irrigation, as a locally administered drug procedure, according to [58,59,100,101] and according to patent documents IN-405074 and JP 2017007978; (3) use a solution containing antioxidants as a water supply for ultrasonic scaling and root planning, according to [62]; (4) recommending toothpaste that contains antioxidants, according to patent documents EP 3445356, CN104666185, and KR 101955468; (5) combining antioxidants with antibiotics for better results, according to patent document RO—133752; and (6) combining antioxidants with conventional periodontal treatments, such as scaling and root planing, according to [59,62,100,101] and according to patent document KR102309837.

Although this work has pointed out that natural antioxidants can be beneficial, it is essential to mention that antioxidants should complement, not replace, conventional and surgical periodontal treatments.

### 3.8. Antioxidant Market: Innovative Solutions in Oral Health

Considering only the alive and granted patents, the syntax (((((PERIODONTAL)/TI/AB/CLMS AND (ANTIOXIDANT)/TI/AB/CLMS)) AND STATE/ACT=ALIVE) AND STATUS/ACT=GRANTED) has selected 132 alive granted patent documents; applying exclusion and inclusion criteria, 74 patents were considered to be analyzed. The granted patents were chosen because they guarantee legal security in licensing patents and the patent’s requirements of novelty/originality, no obviousness, inventive activity, and application. According to the analysis of the number of granted patents related to the region where the assignee was located, the intellectual property (IP) activity is mainly in the United States, Japan, and South Korea. The United States was shown to be the global leader with 27 patent families, followed by South Korea and Japan, each with 17 patent families; the European Union, with 16; and Russia, with 15. 

By analyzing the countries protected by patents, it is possible to visualize the United States, Japan, and South Korea as the target markets, with activity around the technologies related to antioxidants to treat periodontal disease. Furthermore, most companies select countries to file their first patent filings where the research is carried out. However, the top five universities that published the most are not located in the United States, Japan, or South Korea; instead, they are located in England (the University of Birmingham and King’s College London), Italy (Polytechnic University of Marche), Spain (University of Granada), and Turkey (Hacettepe University), presumably because innovative findings of these universities are mainly presented in the form of academic publications. Even if the innovations are presented as patents, universities do not have the expertise to commercialize them [127], representing why companies are the key players. For instance, with a portfolio of 10 inventions, Colgate-Palmolive is the leading player in technology related to antioxidants applied to periodontal disease.

Colgate-Palmolive and the four leading players—named Hybrigenics, in France; KAO, in Japan; Dankook University IACF, and EWA Woman University, both in South Korea—represent 27.02% of the Top 100, supporting the fact that the United States, Japan, and South Korea are the target markets.

The use of antioxidants in healthcare innovation has been a topic of interest and investment across various sectors, ranging from pharmaceuticals and medical devices to wellness and preventive care. Startups are focusing on developing regenerative therapies using antioxidants to enhance healing, particularly in periodontology; it is a promising approach. Biotech startups may work on innovative drug delivery systems that enhance antioxidants’ bioavailability and effectiveness when delivered to specific targets. These antioxidants are essential for treating periodontal disease, because the extracellular matrix of dental biofilm protects microbial cells against chemical and physical insults and hinders the eradication of periodontopathogens in dental plaque [32]. The present study has found that the well-ranked innovative startups are Perio Sciences; FOUR TIGERS, LLC; and Vyome Therapeutics.

This study aimed to compare the intellectual property (IP) market of the analyzed patents with that of graphene batteries, to identify similarities and differences in how IP is monetized across different industries. The markets for antioxidants and graphene are complex, with numerous patents, trademarks, and copyrights. IP is often related to natural compounds, formulations, or extraction methods in the antioxidant market. In contrast, in the graphene market, it can be related to production methods, modifications, or applications of graphene. Companies and research institutions in both industries often compete globally for IP rights, while collaborating on research and development efforts. Research and development are crucial in both markets for innovation and creating new products, processes, or applications. Companies in both markets use various strategies to monetize their IP, such as licensing agreements, joint ventures, or creating proprietary products.

The results have shown that with an estimated value of USD 13.66 M, the antioxidant IP market was considered medium (11% bigger than the graphene battery market), showing a medium volume of expenditure generated by the players. The volume of expenditure generated by the players in one field is a good measure of the technological intensity and maturity of the market. The higher the volume, the bigger the market’s expectations. 

The market strategy index of these technologies was 1.11 and was marketed as a Global Market Strategy, meaning that the technology explored is not protected locally, but globally. 

For Future Market Insights [128], the global periodontal market size in 2022 is expected to expand at a Compound Annual Growth Rate (CAGR) of 10.4%, to reach a valuation of ~USD 24.4 billion by 2032, due to the constantly rising prevalence of periodontal diseases; therefore, the development of devices for the diagnosis and treatment of periodontal diseases is relatively high.

FMI (Future Market Insights) estimated that the antioxidants market size in 2022 was close to USD 2.11 billion, alongside a CAGR of 9.1% from 2023 to 2033. A rise in demand for anti-aging products now drives the global antioxidants market, instead of developing mouthwashes, which are complementary treatments to periodontal disease. On the other hand, while antioxidants are often associated with anti-aging benefits, they can also offer advantages for oral health. Since oral health is interconnected with overall health, investing in antioxidants for oral health may have broader systemic benefits, contributing to overall well-being. Furthermore, the growing importance of oral hygiene is a trend in the periodontal market. The global oral care market is expected to reach USD 54.9 billion by 2026, growing at a CAGR of 3.1% from 2021 to 2026 [129].

### 3.9. Challenges and Limitations of Using Antioxidants to Treat and Prevent Periodontal Disease

Antioxidants have been considered for treating and preventing periodontal disease. However, their effectiveness still needs to be determined, due to various challenges and limitations. These include the inadequate delivery of antioxidants to the periodontal tissues, their potential toxicity, and the variability in the results of different studies. Addressing these challenges is crucial to determining the true potential of antioxidants in periodontal disease management.

One of the main challenges in treating periodontal diseases is ensuring that antioxidants reach the periodontal tissues in sufficient concentrations to have a therapeutic effect. The extracellular matrix of dental biofilm protects microbial cells against chemical and physical insults, making it challenging to eradicate periodontopathogens from dental plaque [32]. Even after undergoing a periodontal pocket cleaning procedure, some subgingival biofilm may remain in the pocket, leading to a persistent chronic inflammatory response that can negatively impact the success of periodontal therapy [9].

It is crucial to emphasize that excessive antioxidant doses can lead to adverse effects. Determining the appropriate dosage of antioxidants for periodontal treatment is a complex process. A low dose may not produce the desired results, while a high dose can result in adverse effects. Therefore, finding a balance and determining the optimal dosage of antioxidants for effective periodontal treatment is essential. Moreover, people may respond differently to antioxidant treatments, due to variations in their metabolism, genetics, and the specific nature of their periodontal disease; the optimal duration of antioxidant treatment for periodontal disease needs to be clearly defined, to establish consistent guidelines for clinical practice. For instance, a randomized controlled trial failed to show any benefit of adding a supplemental dose of vitamin C to nonsurgical periodontal therapy for chronic periodontitis patients [72].

The patent document RO-133752 describes a topical treatment for periodontal lesions that combines antioxidants and antibiotics; the interaction of these compounds, in either a synergistic or antagonistic way, can affect the overall outcome of the treatment.

Future studies must identify particular antioxidants, determine the best ways to deliver them, establish appropriate dosages, and investigate the possible synergistic effects of traditional periodontal treatments. Furthermore, rigorous clinical trials are necessary to evaluate both the short-term and long-term safety and effectiveness of antioxidant-based therapies, before they can be widely adopted for treating periodontal disease.

## 4. Conclusions

This paper discusses a patent mining study focused on using antioxidant phytochemicals in technological advancements to prevent and treat periodontal disease. In fact, oxidative stress is a critical factor in periodontal tissue damage; however, the effectiveness of antioxidant therapy as a standalone treatment for periodontal disease is yet to be established. As a result, conventional periodontal treatments, such as scaling and root planing, remain the main methods of managing periodontal disease, and antioxidant therapy should be considered an adjunct that can improve the clinical response to treatment and the patient outcomes. Antioxidants may be incorporated into the treatment plan to enhance the healing process and minimize the adverse effects of oxidative stress. Antioxidants should be part of periodontal care, as combination therapies can synergistically address multiple aspects of oral health and improve patient outcomes.

The analysis of patent documents has shown that antioxidant phytochemicals, such as polyphenols, carotenoids, and vitamin C, are compounds of interest for developing technological products for oral care. Natural antioxidant can be locally applied to the periodontal tissues using gels, solutions, membranes, dentifrices, chewing gum, orally disintegrating film, mouthwash, mouth spray, or mouth massage cream. Mouthwashes were prominent among the developed technologies. A future perspective is nanotechnology, to enhance the delivery of antioxidants, improving their bioavailability, absorption, and retention. Nanocarriers can target specific sites in the oral environment, such as inflamed periodontal pockets, reducing potential side effects and maximizing the therapy.

The natural origin of these compounds presents a compelling advantage, as they may offer a safer and more sustainable alternative to traditional periodontal therapies, a relevant aspect concerning a promissory market, which, according to this work, is concentrated in the US, China, South Korea, and Japan. As plant-based medicine advances, personalized approaches to periodontal care may emerge, tailoring antioxidant treatments to individual patient needs. This future perspective not only anticipates more effective and targeted therapies for periodontal diseases, but also underscores the importance of exploring nature’s pharmacopeia for innovative solutions in oral health, attracting consumers interested in natural, safe, and environmentally friendly products.

## Figures and Tables

**Table 1:** Quercetin-related patents in periodontal disease.

Title	Description	Assignees
(IN-405074) An oral local drug delivery (ldd) system for preventing and treating periodontal infections	The present invention relates to the drug delivery system for oral care, used in the management of periodontal diseases. Thereby, imparting its antibacterial, anti-inflammatory, and antioxidant activities, which result in the healing of periodontal tissues.	Deepavalli A Nainar Arumuga A Arun Kurumathur Vasudevan
(CN117547509) Folic acid modified quercetin-peppermint oil microemulsion temperature-sensitive gel, preparation method and application thereof	The invention discloses a folic acid-modified, quercetin–peppermint oil, microemulsion temperature-sensitive gel, a preparation method, and applications thereof. The solubility of the quercetin is improved by the peppermint oil, and the folic acid-modified, quercetin–peppermint oil microemulsion can play a role in continuous free radical removal, anti-inflammation, and bone tissue regeneration promotion through targeting macrophages.	ANHUI UNIVERSITY OF CHINESE MEDICINE
(CN114748607) Composition for relieving periodontal tissue diseases and application thereof	The invention discloses a composition for relieving periodontal tissue diseases and applications thereof, and relates to the field of oral care. It plays a role in activating blood and diminishing swelling and inflammation, promoting penetration into a wound to repair a wound surface to better relieve periodontal tissue diseases.	SHANGHAI YUNYAO ORAL MEDICAL TECHNOLOGY
(CN117427034) Injectable antibacterial anti-inflammatory high internal phase emulsion and preparation method and application thereof	The invention relates to an injectable antibacterial, anti-inflammatory, high internal phase emulsion for promoting periodontal tissue regeneration and a preparation method thereof.	SOUTHWEST JIAOTONG UNIVERSITY
(JP2017007978) Oral composition	To provide oral compositions that can inhibit periodontal disease safely, simply, and effectively, without inhibiting antimicrobial properties of flavonoids	SAN EI GEN F F I
(EP2968089) Polyphenol/flavonoid compositions and methods of formulating oral hygienic products	Microemulsions and soluble alkali metal salts of relatively insoluble aglycone polyphenols within oral hygienic products are disclosed for treating oral inflammatory disorders.	VIZURI HEALTH SCIENCES
(US20230381261) A Purified Extract Isolated from Agrimonia Coreana Nakai Containing Abundant Amount of Active Ingredient, the Preparation Thereof, the Composition Comprising the Same as an Active Ingredient for Preventing or Treating Inflammation, Allergy and Atopic Dermatitis and the Use Thereof	(US20230381261) The present invention relates to a purified extract isolated from Agrimonia coreana NAKAI for preventing or treating inflammation, allergy, and atopic dermatitis and the use thereof.	DONGGUK UNIVERSITY
(EP2490543) Extracts, fractions and compositions comprising acetogenins and their applications	The present invention discloses acetogenin(s), extract(s)/fraction(s) derived from Annona squamosa or their compositions for the prevention, treatment, inhibition, or controlling of inflammation- and immune-related diseases or disorders, mediated through cytokines/chemokines or other biomarkers.	LAILA NUTRACEUTICALS
(EP3908297) Fibroblast regenerative cells	Disclosed are compositions, systems, and methods comprising a regenerative fibroblast cell, population, or subsets thereof, possessing regenerative activity useful for the treatment of various degenerative diseases.	FIGENE
(TWI751401) Pharmaceutical synergists for prevention and management of infectious diseases and related chronic diseases and methods thereof	A pharmaceutical composition containing phyto-polyphenols (P), clinical drugs with selective targets (T), and metal ions (M)(Cu2+, SeO32-, Ag+, Mn2+, VO42+, Zn2+, and Sr2+) for use in the prevention and therapy of infectious diseases, neurodegenerative diseases, dementia, diabetes, obesity, metabolic syndromes, periodontitis, dental caries, osteoporosis, cancers, and/or chronic pain.	CHUNG SHAN MEDICAL UNIVERSITY

## Data Availability

Not applicable.
